# Proximate Composition and Nutritional Profile of Rainbow Trout (*Oncorhynchus mykiss*) Heads and Skipjack tuna (*Katsuwonus Pelamis*) Heads

**DOI:** 10.3390/molecules24173189

**Published:** 2019-09-02

**Authors:** Weinan Li, Yu Liu, Wei Jiang, Xiaojun Yan

**Affiliations:** 1 Key Laboratory of Key Technical Factors in Zhejiang Seafood Health Hazards, Institute of Innovation & Application, Zhejiang Ocean University, Zhoushan 316022, China; 2 Laboratory of Seafood Processing, Innovative and Application Institute, Zhejiang Ocean University, Zhoushan 316022, China

**Keywords:** carnosine, anserine, rainbow trout, skipjack tuna, fatty acid, amino acid, by-product

## Abstract

In order to evaluate the application potential of rainbow trout (*Oncorhynchus mykiss*) heads and skipjack tuna (*Katsuwonus pelamis*) heads; proximate composition, amino acids, fatty acids, carnosine, and anserine contents were analyzed in this study. Rainbow trout heads showed significantly higher protein (29.31 g/100 g FW, FW is abbreviation of fresh weight) and lipid (6.03 g/100 g FW) contents than skipjack tuna heads (18.47 g/100 g FW protein and 4.83 g/100 g FW lipid) (*p* < 0.05). Rainbow trout heads and skipjack tuna heads exhibited similar amino acid composition. Essential amino acids constituted more than 40% of total amino acids in both rainbow trout head and skipjack tuna head. The fatty acid profile was different between rainbow trout heads and skipjack tuna heads. Rainbow trout heads mainly contained 38.64% polyunsaturated fatty acids (PUFAs) and 38.57% monounsaturated fatty acids (MUFAs), whereas skipjack tuna heads mainly contained 54.46% saturated fatty acids (SFAs). Skipjack tuna heads contained 4563 mg/kg FW anserine and 1761 mg/kg FW carnosine, which were both significantly higher than those of rainbow trout heads (*p* < 0.05). These results demonstrate that both rainbow trout heads and skipjack tuna heads may be used as materials for recycling high-quality protein. Meanwhile, rainbow trout heads can be used to extract oil with high contents of unsaturated fatty acids, while skipjack tuna heads may be a source for obtaining carnosine and anserine.

## 1. Introduction

Fish is an important food type in people′s lives. During industrial processing, a proportion of the whole fish, which can not be consumed directly, is treated as by-product [1]. Fish by-products contain mainly head, skin, bone, and viscera. At present, only a small part of fish by-products are manufactured as feed ingredients and fertilizer. Most of the by-products are wasted by means of dumping and burying [2,3]. This behavior not only results in the loss of valuable compounds, but also causes environmental and ecological issues. Fish heads constitute the major part of fish by-products, thus how to use them efficiently has received worldwide attention.

Protein is one of the most valuable components in fish heads. Gbogouri et al. prepared protein hydrolysates from Atlantic salmon (*Salmo salar*) heads by enzymatic treatment [4]. Sathivel et al. prepared freeze-dried protein powders from herring (*Clupea harengus*) heads, which exhibited higher emulsifying and fat adsorption capacities than soy protein concentrate [5]. Anissa Haddar et al. extracted and characterized gelation from tuna (*Thunnus thynnus*) heads by hydrolyzing with an alkaline protease [6]. Panpipat and Chaijan recycled protein isolates from bigeye snapper (*Priacanthus tayenus*) heads and evaluated their functional properties [7]. Abdollahi and Undeland investigated the nutritional, structural, functional, and sensorial properties of protein isolates developed from salmon (*S. salar*), cod (*Gadus morhua*), and herring (*C. harengus*) heads, indicating their potential as food ingredients [8]. 

Lipid is another valuable component in fish heads. Glowacz-Rozynska et al. found that oil with low peroxide values was obtained from Atlantic salmon (*S. salar*) heads when the extraction temperature did not exceed 15 °C [9]. Dayse et al. found that enzymatic hydrolysis was a better method for the extraction of oil from yellowfin tuna (*T. albacares*) heads than cooking, pressing, and chemical solvent methods, as the obtained oil showed lower acidity, lower peroxide values, and higher contents of eicosapentaenoic acid (EPA) and docosahexaenoic acid (DHA) [10]. Bruno et al. found that microwave and ultrasound pretreatments improved the extraction yield and quality of oil from rohu (*Labeo rohita*) heads compared to conventional enzymatic extraction [2]. 

**Figure 1:**
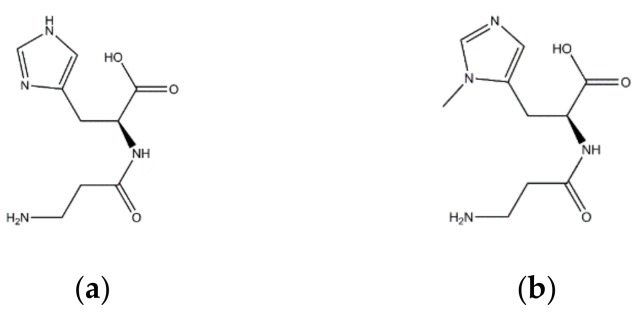
Chemical structure of (**a**) carnosine and (**b**) anserine.

Carnosine (β-alanyl-l-histidine) and anserine (β-alanyl-l-1-methyl-histidine) belong to the family of histidyl dipeptides, whose structures are shown in Figure 1. Many benefits of carnosine and anserine have been revealed, such as antioxidant effects, metal chelating, chaperone and pH-buffering activity, thus they can be potentially applied in many fields [11,12,13,14,15,16]. At present, the industrial application of carnosine and anserine is still restricted, because carnosine and anserine have mainly been derived from chemical synthesis, which results in high price and potential chemical residue. Developing an efficient and low-cost technique for carnosine and anserine synthesis/purification may be a good way to solve this problem. Meanwhile, searching for inexpensive samples with high contents of carnosine or anserine and developing an efficient separation method may be another way to acquire applicable carnosine and anserine. Previous studies have shown that carnosine and anserine were naturally dipeptide in various substances, such as pork, dry-cured hams, chicken meat, rabbit meat, horse meat, beef, and turkey meat [17,18,19,20]. Recently, 1630 mg/kg FW (FW is abbreviation of fresh weight) of anserine was found in salmon (*S. salar*) heads by nuclear magnetic resonance when researchers were investigating metabolites changes during storage [21]. This indicates that fish heads may be good sources for obtaining carnosine and anserine.

The rainbow trout (*Oncorhynchus mykiss*) is a freshwater fish species with worldwide economic and ecological importance [22]. The rainbow trout is mainly obtained through artificial cultivation. After skin-on filleting, about 30% of the whole fish is by-product and currently goes mainly to landfill. The skipjack tuna (*Katsuwonus pelamis*) is a migratory species with a broad distribution from the equatorial to temperate zones [23]. The skipjack tuna is caught mainly to produce canned tuna. During canned tuna manufacturing, about 30% of the whole fish is by-product which is mainly used as fish meal or fertilizer. In this study, the physicochemical properties, amino acid profiles, fatty acid profiles, carnosine and anserine contents of rainbow trout head and skipjack tuna head were analyzed. The aim was to provide foundational data for the utilization of rainbow trout head and skipjack tuna head in future research.

## 2. Results and Discussion

### 2.1. Physicochemical Properties

The moisture, ash, protein, and lipid contents of rainbow trout heads and skipjack tunas head are shown in Table 1. All proximate parameters were significantly different between rainbow trout heads and skipjack tuna heads (*p* < 0.05). The moisture and ash contents of the rainbow trout heads were 64.4 g/100 g FW and 1.91 g/100 g FW, whereas those of the skipjack tuna heads were 75.6 g/100 g FW and 3.88 g/100 g FW, respectively. High content of moisture was also reported in other fish heads, such as Atlantic salmon heads (58.0 g/100 g FW) [24], yellowtail kingfish heads (61.1 g/100 g FW) [24], red salmon heads (65.9 g/100 g FW) [25], and tuna *(T. albacares*) heads (70.1 g/100 g FW) [26]. The ash content refers to the inorganic substance left after being treated at high temperatures for some time. The ash content varies in different kinds of fish heads. For instance, the ash content of tuna (*T. albacares*) heads was 5.18 g/100 FW [25], which was nearly twice higher than that of Atlantic salmon (*S. salar*) heads (2.6 g/100 g FW) [4]. The ash content may be related to the proportion of bones in fish heads [27].

Protein and lipids are both useful nutrients in foods. As shown in Table 1, rainbow trout head contained 29 g/100 g FW protein and 6.0 g/100 g FW lipid, which were significantly higher than those of skipjack tuna head (18 g/100 g FW protein and 4.8 g/100 g FW lipid) (*p* < 0.05). As reported, the protein content varies greatly among different kinds of fish heads. The protein content of Atlantic salmon heads was 23 g/100 g FW [24], while red salmon heads contained only 12 g/100 g FW protein [25]. Protein constituted nearly thirty percent of the fresh weight of rainbow trout heads, which indicated that rainbow trout heads might be a good source for protein extraction. The lipid content was also different in various fish heads. For instance, Atlantic salmon heads and yellowtail kingfish heads contained 20.0 g/100 g FW and 17.3 g/100 g FW lipid, respectively [4,24]. However, Senegalese sole heads and angler (*Lophius piscatorius*) heads had only 2.4 g/100 g FW and 3.2 g/100 g FW of lipids, respectively [28,29]. 

### 2.2. Amino Acid Profile

The amino acid profiles of the rainbow trout heads and skipjack tuna heads are presented in Table 2. Histidine, glycine, and proline contents were significantly different between the rainbow trout heads and skipjack tuna heads (*p* < 0.05). Except for these three amino acids, other amino acids contents of rainbow trout head were similar to those of skipjack tuna head. The lowest content of amino acid was cysteine (<0.4%) in both rainbow trout head and skipjack tuna head, with glutamic acid being the most abundant amino acid. Cysteine was also reported to be the lowest amino acid in herring (*C. harengus*) heads [5]. The top four amino acids in both rainbow trout head and skipjack tuna head were listed as follows: Glutamic acid (15.6% and 16.3%) > aspartic acid (10.3% and 10.9%) > lysine (10.2% and 9.5%) > leucine (8.4% and 8.2%). These four amino acids were also abundant in red salmon heads, Atlantic salmon heads, yellowtail kingfish heads, and herring (*C. harengus*) heads [5,24,30]. Nevertheless, red salmon heads, Atlantic salmon heads, and yellowtail kingfish heads contained the highest content of glycine, which was not a very abundant amino acid in rainbow trout heads nor skipjack tuna heads [4,24]. 

The total contents of essential amino acids in the rainbow trout heads and skipjack tuna heads were 41% and 40%, respectively. These results were in accordance with the contents of essential amino acids of salmon (*S. salar*) by-products (38%) [8] and red salmon heads (37%) [30], but were higher than those of herring (*C. harengus*) heads (31%) [5] and Atlantic salmon heads (23%) [24]. Properties of low-cost, high-protein contents (Table 1) and high essential amino acids contents (Table 2) indicate that rainbow trout heads and skipjack tuna heads may be used as materials for preparing high-quality protein.

### 2.3. Fatty Acid Profile

Fatty acid composition is critical to the application potential of fish oil. Generally, fatty acids are classified into three groups by the number of double bonds in their backbones, namely saturated fatty acids (SFAs), monounsaturated fatty acids (MUFAs) and polyunsaturated fatty acids (PUFAs). Fatty acid profiles of the rainbow trout heads and skipjack tuna heads are presented in Table 3. It is obvious that fatty acid composition is different between rainbow trout heads and skipjack tuna heads. The predominant fatty acids groups of rainbow trout heads were PUFAs (38.6%) and MUFAs (38.6%), which was similar to lipid extracted from Atlantic salmon heads [31]. Nevertheless, SFAs (54.5%) were the predominant fatty acids group in skipjack tuna heads, which was similar to the lipid extracted from golden pompano heads [32].

In both rainbow trout heads and skipjack tuna heads, the major SFAs were palmitic (C16:0), stearic (C18:0), and myristic (C14:0), and the major MUFA was oleic (C18:1). The results were similar to fatty acid profiles of Atlantic salmon heads, yellowtail kingfish heads, and rohu (*L. rohita*) heads [2,24]. 

PUFAs are correlated with the prevention of cardiovascular, dyslipidemias, chronic, and degenerative diseases [33]. As shown in Table 3, PUFAs accounted for 38.8% and 12.7% of the total determined fatty acids in rainbow trout heads and skipjack tuna heads, respectively. From the perspective of sum of PUFAs, lipids from rainbow trout heads are better than skipjack tuna heads. The major PUFA in the rainbow trout heads was linoleic (C18:2 n-6), while the major PUFA in the skipjack tuna heads was docosapentaenoic acid (DHA, C22:6 n-3). Eicosapentaenoic acid (EPA, C20:5 n-3) and DHA are very important PUFAs, because they are beneficial to people‘s health. The EPA and DHA contents of the skipjack tuna heads (1.56% of EPA and 6.3% of DHA) were significantly higher than those of the rainbow trout heads (0.91% of EPA and 2.2% of DHA) (*p* < 0.05). Skipjack tuna heads are more appropriate for EPA and DHA enrichment than rainbow trout heads. As reported, EPA and DHA contents vary in different fish heads. Atlantic salmon heads, yellowtail kingfish heads, tuna (*T. albacares*) heads, and red salmon heads contained more than 10% EPA+DHA, whereas golden pompano heads and rohu (*L. rohita*) heads contained less than 5% EPA+DHA [2,24,25,26,32]. 

### 2.4. Determination of Carnosine and Anserine

#### 2.4.1. Method Performance

In this study, a HPLC method with an ultraviolet detector to determine the values of carnosine and anserine in fish heads was established. A typical HPLC chromatogram of carnosine and anserine is displayed in Supplementary Figure S1A, showing good peak resolution, sharpness, and symmetry. Supplementary Figure S1B gives an example of a rainbow trout head sample. As shown in Table 4, the correlation coefficients between peak areas (Y) and concentrations (X) of carnosine and anserine were 0.9999 and 1, and relative repeatability standard deviation (RSDr) values were 0.62 and 0.51%, respectively. Recoveries were in the range of 92.1–106.1%. Limit of detection (LOD) values of carnosine and anserine were 1.07 and 3.24 mg/kg, and limit of quantitation (LOQ) values were 3.12 and 9.46 mg/kg, respectively. Thus, the established method can meet the requirements for the determination of carnosine and anserine in rainbow trout heads and skipjack tuna heads.

#### 2.4.2. Quantification of Carnosine and Anserine Contents in Rainbow Trout Heads and Skipjack Tuna Heads

**Figure 2:**
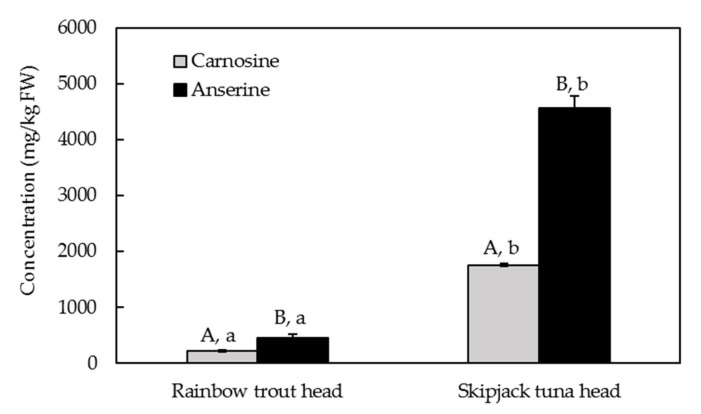
The contents of carnosine and anserine in rainbow trout heada and skipjack tuna heada. Different lowercase letters indicate significant differences between rainbow trout heads and skipjack tuna heads of the same substance (*p* < 0.05), while different capital letters indicate significant differences between carnosine and anserine of the same fish head (*p* < 0.05). FW: fresh weight of rainbow trout head or skipjack tuna head.

Carnosine and anserine contents of rainbow trout heads and skipjack tuna heads are presented in Figure 2. On the one hand, both rainbow trout heads and skipjack tuna heads contained significantly higher contents of anserine than carnosine (*p* < 0.05). On the other hand, skipjack tuna heads contained 4563 mg/kg FW anserine and 1761 mg/kg FW carnosine, which were significantly higher than those of rainbow trout heads (*p* < 0.05). Previous studies have shown that pig meat had 3071–3632 mg/kg FW carnosine and 1869–2359 mg/kg anserine, while chicken meat had 200–1600 mg/kg FW carnosine [17,19]. Recently, 1630 mg/kg FW anserine was reported in salmon (*S. salar*) heads [21]. The results indicate that skipjack tuna heads may be a good source for carnosine and anserine extraction, because of their low price and high contents of target substances.

## 3. Materials and Methods

### 3.1. Chemicals

Anserine, carnosine, fatty acid methyl ester standard mixture, methyl heneicosanoate reference standard, o-phthaldialdehyde, and amino acid standard mixture were purchased from Sigma-Aldrich Chemical Co. (St. Louis, MI, USA). Methanol of chromatographic grade was purchased from Merck (Darmstadt, Germany). Ultrapure water was prepared by Milli-Q (Millipore, Billerica, MA, USA). Other chemicals of analytical reagent grade were purchased from Sinopharm Group (Shanghai, China). 

### 3.2. Samples

The rainbow trout heads and skipjack tuna heads used in this study were provided by two aquatic companies. The rainbow trout was obtained through artificial cultivation and was about 2.5 years old. The weight of the rainbow trout was in the range of 2.0–2.5 kg. The skipjack tuna was caught from ocean, with the weight ranging from 2.5 to 4.5 kg. Samples were transferred to the laboratory with ice and were kept at −80 °C in a refrigerator from Haier Group (DW−86L388A, Qingdao, China). Both rainbow trout head and skipjack tuna head samples were thawed at 4 °C. To ensure the sample representativity, twenty heads of each fish species were minced to uniformity by a high speed tissue masher (DS-1, Shanghai, China) and then stored at −20 °C until use.

### 3.3. Determination of Physicochemical Properties

Moisture and ash contents were determined by thermal treatment at different temperatures. Five grams of each sample was placed onto a crucible, and was dried in an electro-thermostatic clast oven (GZX-9140MBE, Shanghai, China) at 105 °C for 6 h. Moisture content (g/100 g FW) was calculated as follows: (weight of the sample before drying-weight of the sample after drying)/weight of the sample before drying × 100. The sample after drying was further treated in a chamber furnace (KSL-1200X-J, Hefei, China) at 550 °C for 4 h. Ash content (g/100 g FW) was calculated as follows: weight of the sample after high temperature treatment/weight of the sample before drying ×100.

Crude protein content was determined by a Kjeldahl method containing two steps. Firstly, 0.2 g of sample was added to a digestion tube with one piece of a Kjeldahl tablet and 10 mL of concentrated sulfuric acid. The digestion tube was heated in a graphite digestion apparatus (SH220N, Jinan, China) at 300 °C for 0.5 h and 380 °C for 1.5 h, until a transparent blue solution was obtained. Secondly, crude protein content (g/100 g FW) of the sample was determined by an automatic Kjeldahl apparatus (Hanon K9860, Jinan, China), with a protein conversion factor of 6.25.

Crude lipid content was determined by the Soxhlet solvent extraction method, using petroleum ether as the solvent. Five grams of the sample was dried in an electro-thermostatic clast oven (GZX-9140MBE, Shanghai, China) at 105 °C for 6 h. Then, the crude fat content (g/100 g FW) of the sample was determined by an automatic fat analyzer (Hanon SOX406, Jinan, China) at 60 °C for 5 h.

### 3.4. Determination of Amino Acid Profile

Samples were dried by a vacuum freeze drier (FD-1C-80, Shanghai, China). Dried samples were digested using 6 mol/L HCl at 110 °C for 24 h under nitrogen atmosphere, and pre-column derivatization with o-phthaldialdehyde was carried out accordingly [34]. Amino acid composition was assayed by reversed phase-high performance liquid chromatography (RP-HPLC) in an Agilent 1100 system equipped with quaternary pump, column oven, ultraviolet detector, and automatic sampler [35] with some modifications. A reversed-phase chromatographic column (ODS HYPERSIL C18, 250 mm × 4.6 mm i.d., 5 μm) was purchased from Thermo Fisher Scientific (Waltham, MA, USA). The mobile phase A (pH 7.2) was 60 mmol/L sodium acetate/triethylamine/tetrahydrofuran (500:0.12:2.5, *v*/*v*/*v*), while the mobile phase B (pH 7.2) was 150 mmol/L sodium acetate/methanol/acetonitrile (1:2:2, *v*/*v*/*v*). They were filtrated through 0.22 μm filter and degassed ultrasonically prior to use. The chromatographic column was equilibrated at 40 °C. The elution gradient was set as follows: 0–27.5 min, 1.0–1.0 mL/min, 92–40% mobile phase A; 27.5–31.5 min, 1.0–1.5 mL/min, 40–0% mobile phase A; 31.5–32 min, 1.5–1.5 mL/min, 1–0% mobile phase A; 32–34 min, 1.5–1.0 mL/min, 0–100% mobile phase A; 34–35.5 min, 1.0–1.0 mL/min, 100–92% mobile phase A. The derivatized amino acids were detected by the ultraviolet detector at 338 nm. The injection volume was 10 μL. A known mixture of different amino acids was applied as an external standard. All amino acids were identified by comparing their retention time with those of the amino acid reference standards. Calibration curves were constructed of peak area versus standard amino acid concentration. The content of each amino acid was expressed as % of total determined amino acids.

### 3.5. Determination of Fatty Acid Profile

Samples were dried by a vacuum freeze drier (FD-1C-80, Shanghai, China). Dried samples were methyl esterified according to the American Oil Chemists’ Society (AOCS) official method [36]. Fatty acid composition was determined by a gas chromatography system in a Shimadzu Model GC-2030 Nexis system, which was equipped with a capillary gas chromatography column (TR-FAME 60 m × 0.25 mm i.d. × 0.25 μm) and a flame ionization detector [37]. The gas chromatography analysis protocols were briefly summarized as follows: the initial oven temperature was maintained at 130 °C for 3 min before it was increased to 200 °C by 5 °C/min and held for 10 min, then increased to 220 °C at the rate of 2 °C/min, and kept for 3 min. The temperatures of the injector and flame ionization detector were set at 250 and 280 °C, respectively. The total analysis procedure lasted for 40 min. Constant carrier gas (N_2_) flow was set as 1.8 mL/min, the split ratio was 100, and the injection volume was 1 μL. All fatty acids were identified by comparing their retention time with those of the fatty acid methyl ester reference standards. The content of each fatty acid was calculated using methyl heneicosanoate as an internal standard. The content of each free fatty acid was expressed as % of total determined fatty acids.

### 3.6. Determination of Carnosine and Anserine Content

Both carnosine and anserine in the samples were extracted by water. Briefly, a 1.0 g portion of the sample was mixed with 9.0 mL of ultrapure water by a vortex mixer (XW-80A, Shanghai, China). The mixture was heated at 50 °C for 10 min in an electro-thermostatic water bath (HH-1, Jiangsu, China), and then was centrifuged at 10000 g and 4 °C for 10 min. Each sample was extracted twice by the above procedures. The supernatants were collected and diluted with water to 25 mL. The obtained solution was filtered through a 0.22 μm filter (Millipore, Bedford, MA, USA) prior to analysis.

The contents of carnosine and anserine in samples were determined by the HPLC method. The HPLC system consisted of a Waters e2695 series (Medford, MA, USA) equipped with a degasser, quaternary pump, column oven, ultraviolet detector, and automatic sampler. A reversed-phase chromatographic column (Capcell PAK C18 AQ S5, 250 mm × 4.6 mm i.d., 5 μm) was purchased from Osaka Soda Co. (Osaka, Japan). The mobile phase, consisting of 99% phosphate buffer solution (20 mmol/L, pH 9.0) and 1% methanol, was filtrated through a 0.22 μm filter and degassed ultrasonically prior to use. The chromatographic column was equilibrated at 35 °C. The flow rate was set at 1.0 mL/min. The injection volume was 5 μL. Carnosine and anserine were detected by the ultraviolet detector at 230 nm. Calibration curves were constructed of peak areas versus standard carnosine and anserine concentrations (1.0–500 mg/L). The limit of detection (LOD), the limit of quantification (LOQ), and the recovery and the relative repeatability standard deviation (RSDr) were calculated according to Snyder et al. [38]. LOD and LOQ were estimated as follows: LOD = 3.3 σ/S’ and LOQ = 10 σ/S’, where σ and S‘ are the standard deviation and slope of the calibration curve, respectively.

### 3.7. Statistical Analysis

Statistical analyses were performed using the software IBM SPSS Statistics 22.0 (IBM, Armonk, NY, USA). Data were analyzed by one-way analysis of variance (ANOVA) with the Tukey test. All the measurements were carried out in triplicate and the data were expressed as average ± standard deviation.

## 4. Conclusions

This study investigated proximate composition, amino acids, fatty acids, and carnosine and anserine contents of two fish by-products, namely rainbow trout heads and skipjack tuna heads. The results provided foundation data for their utilization. Both rainbow trout heads and skipjack tuna heads are good sources for protein isolating because of their high contents of protein and high proportions of essential amino acids. Rainbow trout heads can be used to extract oil because of their abundant unsaturated fatty acids. Skipjack tuna heads were a good source for obtaining carnosine and anserine because of its low price, and high anserine and carnosine contents. In future, an overall extraction technique for protein, oil, carnosine, and anserine should be developed for high-value utilization of rainbow trout heads and skipjack tuna heads.

## Figures and Tables

**Table 1:** Physicochemical properties of rainbow trout heads and skipjack tuna heads (g/100 g FW).

Parameters	Rainbow Trout Heads	Skipjack Tuna Heads
Moisture	62.4 ± 0.7 ^a^	75.6 ± 0.5 ^b^
Ash	1.91 ± 0.06 ^a^	3.88 ± 0.08 ^b^
Protein	29 ± 1 ^b^	18 ± 3 ^a^
Lipid	6.0 ± 0.3 ^b^	4.8 ± 0.5 ^a^

FW: fresh weight of rainbow trout heads or skipjack tuna heads. Values in the same line followed by different letters differ significantly (*p* < 0.05).

**Table 2:** Amino acid profiles of rainbow trout heads and skipjack tuna heads (% of total determined amino acids).

Amino Acids	Rainbow Trout Heads	Skipjack Tuna Heads
Aspartic acid	10.3± 0.8 ^a^	10.9 ± 0.6 ^a^
Glutamic acid	15.6 ± 0.8 ^a^	16.3 ± 0.9 ^a^
Serine	3.3 ± 0.1 ^a^	3.7 ± 0.3 ^a^
Histidine	7.3 ± 0.2 ^b^	2.9 ± 0.2 ^a^
Glycine	4.8 ± 0.2 ^a^	6.2 ± 0.2 ^b^
Threonine	3.8 ± 0.1 ^a^	3.9 ± 0.2 ^a^
Arginine	6.2 ± 0.3 ^a^	6.3 ± 0.1 ^a^
Alanine	6.0 ± 0.4 ^a^	6.6 ± 0.2 ^a^
Tyrosine	2.4 ± 0.1 ^a^	2.4 ± 0.1 ^a^
Cysteine	0.38 ± 0.04 ^a^	0.24 ± 0.11 ^a^
Valine	6.2 ± 0.3 ^a^	6.1 ± 0.2 ^a^
Methionine	2.7 ± 0.1 ^a^	2.6 ± 0.3 ^a^
Phenylalanine	4.2 ± 0.4 ^a^	4.4 ± 0.6 ^a^
Isoleucine	5.6 ± 0.3 ^a^	5.4 ± 0.3 ^a^
Leucine	8.4 ± 0.4 ^a^	8.2 ± 0.2 ^a^
Lysine	10.2± 0.7 ^a^	9.5 ± 0.4 ^a^
Proline	2.7 ± 0.3 ^a^	4.5 ± 0.2 ^b^
∑ *EAAs*	41	40

∑ *EAAs*: sum of essential amino acids. Values in the same line followed by different letters differ significantly (*p* < 0.05).

**Table 3:** Fatty acid profiles of rainbow trout heads and skipjack tuna heads (% of total determined fatty acids).

Fatty Acids	Rainbow Trout Heads	Skipjack Tuna Heads
C12:0	0.03 ± 0.01 ^a^	0.09 ± 0.00 ^b^
C14:0	1.65 ± 0.02 ^a^	4.70 ± 0.03 ^b^
C15:0	0.20 ± 0.01 ^a^	1.38 ± 0.03 ^b^
C16:0	15.4 ± 0.2 ^a^	36 ± 1 ^b^
C17:0	0.22 ± 0.03 ^a^	1.69 ± 0.06 ^b^
C18:0	4.6 ± 0.1 ^a^	10.0 ± 0.2 ^b^
C20:0	0.23 ± 0.04 ^a^	0.60 ± 0.03 ^b^
C22:0	0.49 ± 0.02 ^b^	0.37 ± 0.02 ^a^
∑ *SFAs*	22.8	54.5
C16:1	3.13 ± 0.05 ^a^	6.8 ± 0.3 ^b^
C18:1	32 ± 1 ^b^	23.9 ± 0.8 ^a^
C20:1 n-9	1.7 ± 0.1 ^a^	1.9 ± 0.1 ^a^
C22:1 n-9	1.74 ± 0.08 ^b^	0.26 ± 0.01 ^a^
∑ *MUFAs*	38.6	32.8
C18:2 n-6	29.1 ± 0.6 ^b^	0.98 ± 0.01 ^a^
C18:3 n-6	0.35 ± 0.06 ^b^	0.11 ± 0.03 ^a^
C18:3 n-3	3.1 ± 0.2 ^b^	0.48 ± 0.05 ^a^
C20:2 n-6	1.20 ± 0.03 ^b^	0.35 ± 0.02 ^a^
C20:3 n-3	0.27 ± 0.03 ^b^	0.10 ±0.01 ^a^
C20:3 n-6	0.49 ± 0.04 ^b^	0.08 ± 0.00 ^a^
C20:4 n-6	0.37 ± 0.01 ^a^	1.56 ± 0.04 ^b^
C20:5 n-3 (EPA)	0.91 ± 0.06 ^a^	1.28 ±0.20 ^b^
C22:3 n-3	0.06 ± 0.01 ^a^	0.20 ± 0.06 ^b^
C22:4 n-3	0.24 ± 0.05 ^b^	0.10 ± 0.00 ^a^
C22:4 n-3	0.07 ± 0.00 ^a^	0.80 ± 0.02 ^b^
C22:5 n-3	0.30 ± 0.07 ^a^	0.37 ± 0.03 ^a^
C22:6 n-3 (DHA)	2.2 ± 0.1 ^a^	6.3 ± 0.1 ^b^
∑ *PUFAs*	38.6	12.7

∑ *SFAs*: sum of saturated fatty acids. ∑ *MUFAs*: sum of monounsaturated fatty acids. ∑ *PUFAs*: sum of polyunsaturated fatty acids. Values in the same line followed by different letters differ significantly (*p* < 0.05).

**Table 4:** Validation data for carnosine and anserine in rainbow trout heads and skipjack tuna heads by HPLC method.

	Carnosine	Anserine
Regression equation *	Y = 1303.9X + 1838.2	Y = 3002.9X − 1190.7
*R* ^2^	0.9999	1
RSDr (%, n = 10)	0.62	0.51
Recovery (%), fortified with 0.5 mg/g	106.1	106.0
Recovery (%), fortified with 1.0 mg/g	103.6	92.7
Recovery (%), fortified with 1.5 mg/g	97.8	92.1
LOQ (mg/kg)	3.12	9.46
LOD (mg/kg)	1.07	3.24

* Dynamic range was evaluated within 1.0 and 500 mg/L for both compounds. Y: peak areas. X: carnosine or anserine concentrations. RSDr: relative repeatability standard deviation. LOD: limit of detection. LOQ: limit of quantitation.

## Supplementary Materials

The following are available online, Figure S1. Typical HPLC chromatograms of (A) standard solution (250 mg/L) and (B) the extract of rainbow trout head.
