# Supplementary material for: Proximate Composition and Nutritional Profile of Rainbow Trout (Oncorhynchus mykiss) Heads and Skipjack tuna (Katsuwonus Pelamis) Heads
Source: Molecules. 2019 Sep 2;24(17):3189. doi: 10.3390/molecules24173189 (PMC6749204; doi:10.3390/molecules24173189)
Supplement: Supplementary file 1 [file molecules-24-03189-s001.pdf]

Supplementary File

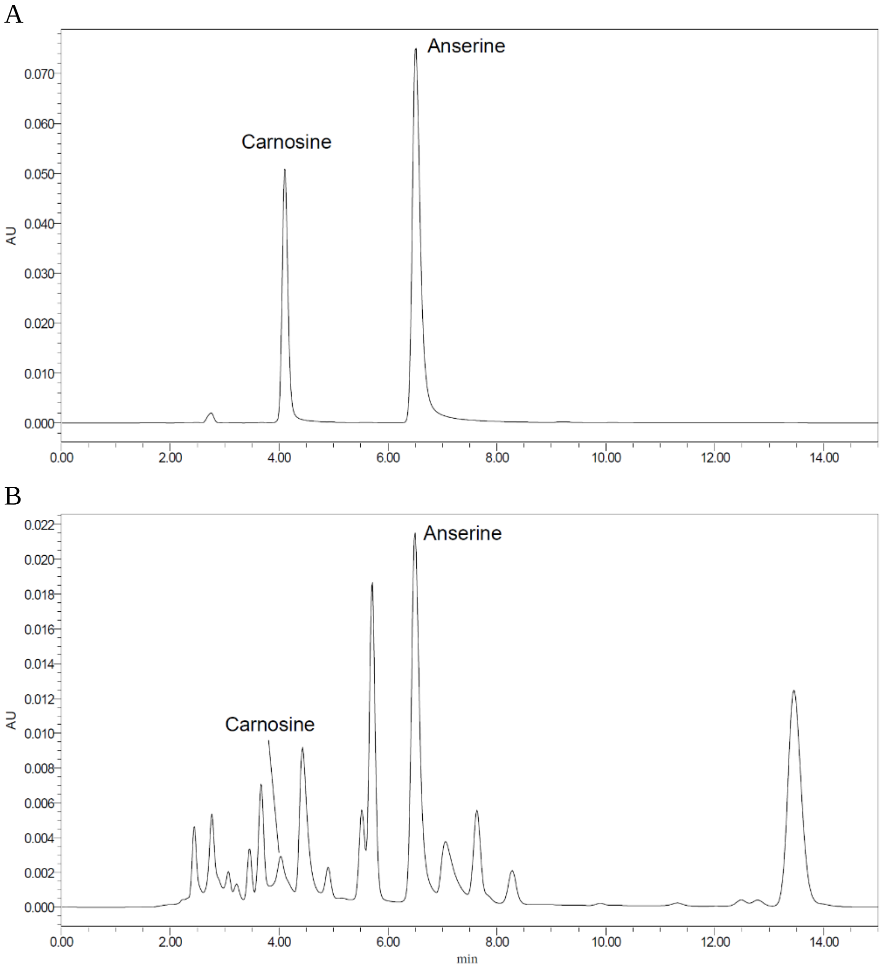

Figure S1. Typical HPLC chromatograms of (A) standard solution (250 mg/L) and (B) the extract of rainbow trout head.
